# A Placebo-Controlled, Pseudo-Randomized, Crossover Trial of Botanical Agents for Gulf War Illness: Curcumin (*Curcuma longa),* Boswellia (*Boswellia serrata*), and French Maritime Pine Bark (*Pinus pinaster*)

**DOI:** 10.3390/ijerph18052468

**Published:** 2021-03-03

**Authors:** Emily K. Donovan, Sophia Kekes-Szabo, Joanne C. Lin, Rebecca L. Massey, James D. Cobb, Kathleen S. Hodgin, Timothy J. Ness, Carl Hangee-Bauer, Jarred W. Younger

**Affiliations:** 1 Department of Psychology, Virginia Commonwealth University, White House, 806 West Franklin Street, Richmond, VA 23284, USA; donovanek@mymail.vcu.edu; 2 Department of Psychology, Vanderbilt University, PMB 407817, 2301 Vanderbilt Place, Nashville, TN 37240, USA; sophia.kekes-szabo@vanderbilt.edu; 3 School of Pharmacy, University of Auckland, 85 Park Road, Grafton, Auckland 1023, New Zealand; joanne.lin@auckland.ac.nz; 4 UAB School of Medicine, University of Alabama at Birmingham, 1670 University Blvd, Birmingham, AL 35223, USA; rlmassey@uab.edu; 5 Department of Psychology, University of Alabama at Birmingham, CH 233, 1300 University Blvd, Birmingham, AL 35233, USA; cobb.james1894@gmail.com (J.D.C.); kathleenhodgin@uabmc.edu (K.S.H.); 6 Department of Anesthesiology and Perioperative Medicine, University of Alabama at Birmingham, BMR2-208, 901 19th St. S, Birmingham, AL 35205, USA; tness@uabmc.edu; 7 San Francisco Natural Medicine, 1615 20th Street, San Francisco, CA 94107, USA; carl@sfnatmed.com

**Keywords:** boswellia, curcumin, maritime pine

## Abstract

This report is part of a larger study designed to rapidly and efficiently screen potential treatments for Gulf War Illness (GWI) by testing nine different botanicals. In this placebo-controlled, pseudo-randomized, crossover clinical trial of 20 men with GWI, we tested three botanical agents with putative peripheral and central anti-inflammatory actions: curcumin (*Curcuma longa*), boswellia (*Boswellia serrata*), and French maritime pine bark extract (*Pinus pinaster*). Participants completed 30 +/− 3 days of baseline symptom reports, followed by 30 +/− 3 days of placebo, 30 +/− 3 days of lower-dose botanical, and 30 +/− 3 days of higher-dose botanical. Participants then repeated the process with a new botanical until completing up to three botanical cycles. Data were analyzed using linear mixed models. Curcumin reduced GWI symptom severity significantly more than placebo at both the lower (*p* < 0.0001) and higher (*p* = 0.0003) dosages. Boswellia was not more effective than placebo at reducing GWI symptoms at either the lower (*p* = 0.726) or higher (*p* = 0.869) dosages. Maritime pine was not more effective than placebo at the lower dosage (*p* = 0.954) but was more effective than placebo at the higher dosage (*p* = 0.006). This study provides preliminary evidence that curcumin and maritime pine may help alleviate symptoms of GWI. As a screening study, a final determination of the efficacy of these compounds for all individuals with GWI cannot be made, and further studies will need to be conducted to determine strength and durability of effects, as well as optimal dosage. These results suggest that GWI may, at least in part, involve systemic inflammatory processes. This trial was registered on ClinicalTrials.gov (NCT02909686) on 13 September 2016.

## 1. Introduction

Gulf War Illness (GWI) is a chronic, multisymptom condition of unknown etiology [1]. There are currently no FDA-approved treatments for the approximately 175,000 to 250,000 active sufferers [2], and there is no consensus on effective off-label treatments.

While many pathophysiological mechanisms of GWI are currently being explored [3], several studies suggest dysregulated immune functioning is involved [4,5,6]. We have previously reported interleukin (IL)-1beta to be elevated on days when GWI symptom severity is highest [7]. Additionally, plasma C-reactive protein (CRP) and brain-derived neurotrophic factor (BDNF) have been found to be significantly elevated compared to a control group of healthy Veterans [8]. The wide variety of symptoms experienced by those with GWI (i.e., pain, fatigue, cognitive dysfunction, gastrointestinal issues, respiratory difficulties, and dermatological complaints) have led some researchers to suggest that GWI is a neuroimmune condition involving systemic inflammation [9].

Because GWI involves a discrete, aging cohort of Veterans, there is a shrinking window of time to produce treatments that will improve overall GWI symptoms for those living with this illness. Therefore, it is important to rapidly screen potential treatments in order to prioritize agents for efficacy testing. One method for screening agents rapidly is to test several candidate treatments serially in a cohort of participants. In this human in vivo study, we examined the effects of nine different botanicals with known peripheral and/or central anti-inflammatory actions. Three of the tested agents (curcumin, boswellia, and maritime pine) were grouped together prior to analyses to be discussed in this report, and findings from the other six agents will be presented in two separate manuscripts.

Curcumin is a compound in turmeric with reported anti-inflammatory, microglia-modulating, and neuroprotective properties [10,11]. Curcumin reduces the release of nitric oxide (NO) [12], tumor necrosis factor (TNF)-alpha [12,13], IL-6 [13], IL-1beta [13], reactive oxygen species (ROS) and monocyte chemoattractant protein (MCP)-1 [14], and macrophage inflammatory protein (MIP)-1alpha [15]. In an animal model of GWI, rats treated with daily curcumin therapy for 30 days displayed greater cognitive and mood function, enhanced neurogenesis, and diminished inflammation and mitochondrial dysfunction 60 days after treatment concluded [16]. Additionally, curcumin protects neurons from infection-associated neurotoxicity [17], as well as from inflammation-associated cognitive deficits (i.e., memory dysfunction [18]). Because of its high probability of blood–brain barrier penetration and its suppressive effects on microglia [19], curcumin is currently being considered as a treatment for various central nervous system disorders. These disorders include delirium [20], Alzheimer’s Disease [21], and central pain sensitization in musculoskeletal disorders such as monoarthritis [15].

Boswellia (Indian frankincense), an extract of the *Boswellia serrata* tree with a long history of religious, cultural, and medicinal use in Asia and Africa [22,23,24], has been shown to have potent anti-inflammatory properties [25]. In an in vitro study, boswellia reduced inflammatory damage of the colonic epithelial cells, suppressing interferon (IFN)-gamma and TNF-alpha [26]. In animals, boswellia demonstrated neuroprotective and anti-inflammatory actions in a model of stroke [27] and significantly reduced levels of inflammatory mediators in a model of arthritis [28]. In patients with chronic kidney disease, boswellia reduced levels of IL-6 [29].

French maritime pine bark extract has been demonstrated to be neuroprotective in an animal model of traumatic brain injury [30] via reduction of thiobarbituric acid reactive species. The agent reduced TNF-alpha and IL-1beta expression in a mouse model of Parkinson’s Disease [31]. It also reduced the production of TNF-alpha, IL-6, and IL-1beta from lipopolysaccharide-stimulated mouse microglia [32]. In humans, it was shown to reduce CRP [33] and IL-1beta [34] in patients with osteoarthritis. The anti-inflammatory effects of this agent are suggested to result from agonistic toll-like receptor (TLR) activity (particularly TLR1/2 and TLR 2/6), inducing production of IL-10 [35].

The aim of this study was to identify the most promising anti-inflammatory botanical compounds for the treatment of GWI symptoms. Each of the three botanical agents was tested in a lower-dosage and higher-dosage condition against both baseline and placebo. We hypothesized that both lower and higher dosages of these agents would lead to significantly decreased GWI symptom severity compared to baseline and placebo.

## 2. Materials and Methods

The full study was a pseudo-randomized, placebo-controlled, crossover clinical trial testing the effects of nine different anti-inflammatory botanical compounds on symptoms of GWI. The list of agents tested in the larger study were curcumin, boswellia, maritime pine, epimedium, fisetin, luteolin, reishi mushroom, resveratrol, and stinging nettle. Participants trialed the botanicals serially, with each participant receiving up to three botanical agents. Outcome measures were obtained daily throughout the entire duration of participation. The University of Alabama at Birmingham (UAB) Institutional Review Board first approved the study protocol (F150318011) on 30 June 2015, and the study was registered on ClinicalTrials.gov (NCT02909686) on 21 September 2016. Participants were recruited to the study through public flyers and online postings. All participants were given a written description of the study and provided written informed consent. Participants were randomized to botanicals so that each condition was completed by at least 10 individuals.

### 2.1. Participants

Men were considered for inclusion in the study if they were aged 37–65 and able to attend up to 11 study visits every 30 +/− 3 days. Participants were required to meet Kansas GWI case definition criteria [36], with the exception that (1) participants were permitted to have a comorbid diagnosis of diabetes mellitus type 2 if controlled with medications and if having a hemoglobin A1C ≤ 9% and (2) one individual with a remote history of cancer (Hodgkin’s lymphoma in remission for 20 years) was included. Participants must have been present in the Persian Gulf between August 1990 and August 1991.

Exclusionary criteria included current opioid, daily anti-inflammatory, nitroglycerine, or lithium medication use; history of anaphylaxis to study botanical compounds; presence of severe depressive symptoms as indicated by a Hospital Anxiety and Depression Scale (HADS [37]) depression subscale score ≥16; presence of a blood or clotting disorder; hypotension (under 90/60 mmHg) or history of cardiovascular disease; diagnosed rheumatologic or autoimmune disease; and acute infection (body temperature over 100.4 °F). Baseline laboratory values of erythrocyte sedimentation rate (ESR) > 40 mmHr, positive rheumatoid factor (RF), CRP > 10.0 mg/L, or positive antinuclear antibody (ANA) were also exclusionary. Participants could also not have current Posttraumatic Stress Disorder (PTSD). PTSD was initially screened with the PTSD Checklist—Military Version (PCL-M [38]), and individuals with PCL-M scores ≥50 were given the Clinician Administered PTSD Scale (CAPS-5 [39]) for a final determination.

### 2.2. Botanicals

The botanical compounds were sourced from a university-approved vendor, Pure Encapsulations (Sudbury, MA, USA), prior to being sent to a compounding pharmacy for re-encapsulation. Botanical compounds were re-encapsulated in size 0 or size 00 blue gelatin capsules by Double Oak Mountain Pharmacy in Birmingham, AL. Capsules were placed in standardized QUBE Weekly (28 cavity) Cold Seal Compliance Blister packs (Pharmacy Automation Supplies, Romeoville, IL, USA).

Curcumin was obtained as Pure Encapsulation’s CurcumaSorb product (SKU#: MCU1), which contains the trademarked Meriva^®^ turmeric phytosome (Indena, S.p.A., Milan, Italy). Curcumin was administered at 1000 mg/day (lower dosage condition) and 4000 mg/day (higher dosage condition). Boswellia was obtained as Pure Encapsulation’s Boswellia product (SKU# BW31) and was administered at 400 mg/day and 800 mg/day. Maritime pine bark extract was obtained as Pure Encapsulation’s Pycnogenol (pine bark extract) product (SKU# PY16) which contains the trademarked Pycnogenol (Horphag Research, Hoboken, NJ, USA). Maritime pine was administered at 200 mg/day and 400 mg/day. All botanicals were administered twice per day (morning and evening), with the total daily dosage being split evenly between the morning and evening doses.

### 2.3. Study Protocol

All participants were assessed for initial eligibility for the study using an online prescreening questionnaire, followed by a phone interview. Individuals who met initial inclusion criteria after the online screening and phone interview were given an in-person screening at UAB’s Center for Clinical and Translational Science Clinical Research Unit (CCTS CRU).

At the in-person screening visit, participants provided written informed consent as well as blood samples to test for exclusionary lab values. Participants also completed baseline study measures on a tablet device and were loaned a tablet for completion of daily symptom reporting during the study. Following the screening visit, participants reported symptoms every day (in the evening) during a one-month (30 +/− 3 days) baseline period, which served as a habituation period and a means to assess whether participants would reliably complete their daily symptom reports. Participants were allowed to continue with study participation if they completed at least 80% of baseline symptom reports.

**Figure 1:**
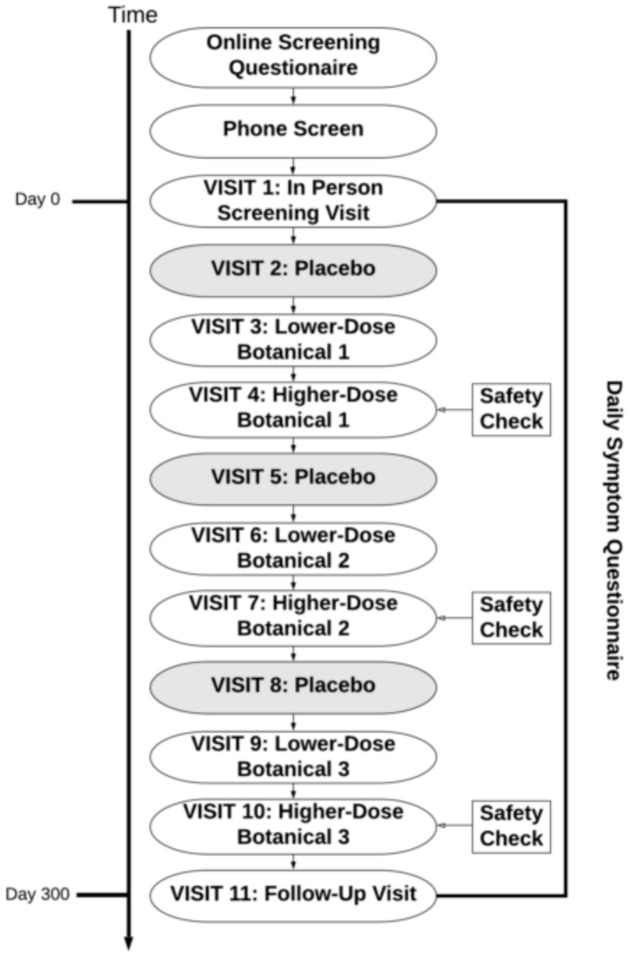
Study protocol. Each participant completed testing of up to three botanicals. Some participants opted to re-enroll in the study protocol after completion, resulting in a maximum of six botanical assignments. For each botanical, there was a placebo condition, followed by lower-dose botanical and higher-dose botanical conditions. The period of time between visits was 30 +/− 3 days.

Participants were then pseudo-randomized to receive up to three out of the nine botanical compounds, in the design presented in Figure 1. Botanical assignments were pseudo-randomized so that (1) approximately equal numbers of participants would take each botanical and (2) to ensure there were no drug interactions that contraindicated the use of the botanicals assigned to participants. There were no contraindications used for curcumin or boswellia. Individuals with prediabetes, evidence of diabetes, or diabetic medications were excluded from taking maritime pine due to its lowering effects on blood glucose [40]. Contraindications were handled by a pharmacist with no other connections to the study so that all research personnel could remain blinded to the botanical being assigned. After completion of a full protocol of three botanical assignments, participants were offered the opportunity to re-enroll into the study protocol to receive up to three more botanicals.

After the baseline period, participants returned once monthly (every 30 +/− 3 days) for up to 10 additional visits to the CCTS CRU for dispensation of their placebo or botanical capsule kits. At visits 4, 7, and 10, participants received additional blood draws to monitor liver and kidney function (sodium, potassium, chloride, bicarbonate, anion gap, glucose, blood urea nitrogen (BUN), creatinine, calcium, phosphorus, albumin, total protein, total bilirubin, direct bilirubin, indirect bilirubin, alkaline phosphatase, aspartate aminotransferase (AST), and alanine aminotransferase (ALT)). Participants were compensated for each laboratory visit.

For each botanical, participants followed the same protocol: one month (30 +/− 3 days) of placebo, one month (30 +/− 3 days) of lower-dose botanical, and one month (30 +/− 3 days) of higher-dose botanical. Study participants were blinded to both the assigned botanicals and administration protocol (placebo, lower-dose, higher-dose). This administration order was chosen for safety reasons, i.e., adverse effects could be detected in a lower-dose condition before a higher-dose, with possibly greater adverse effects, would be administered. Research personnel were blinded to the assigned botanicals but not to the administration protocol. The study pharmacist was unblinded to both administration protocol and assigned botanicals but had no contact with study participants. Adherence to botanicals was checked at each study visit when participants returned their used blister packs. If participants had missed doses, study staff reminded them of the importance of a regular dosing schedule.

### 2.4. Screening Measures

The Kansas GWI case definition [36] was used to determine if participants met criteria for GWI. Exclusionary criteria for the definition included certain chronic conditions (heart disease, stroke, lupus, multiple sclerosis, cancer (other than skin cancer), melanoma, and liver disease) not associated with service in the Gulf War, as well as certain conditions that could impact participants’ ability to report their symptoms (bipolar disorder or manic depression, schizophrenia, or recent hospitalization for alcohol or drug dependence, depression, or PTSD). Inclusionary criteria for the definition required that participants report presence of symptoms that began during or after service in the Gulf War. Each symptom was scored on a severity scale of 0 to 3, with 0 = none and 3 = severe. Veterans with a score of 2 (at least one moderate symptom or two mild symptoms) or greater in at least three out of six symptom domains (fatigue, pain, neurological/cognitive/mood, skin, gastrointestinal, and respiratory) met criteria for GWI.

The HADS [37] was used to screen for severe depressive symptoms. Potential participants completed the questionnaire via the Qualtrics Research Suite Online Application as part of the online prescreening process. The HADS consists of 14 items divided into two, seven-item subscales: Anxiety (HADS-A) and Depression (HADS-D). Respondents rate items on a 0 to 3 scale, with higher ratings indicating greater presence of the symptoms. Five of the 14 items are reverse scored. The total score is calculated by summing all items and ranges from 0–42. Scores of 16 or greater on the HADS-D subscale during prescreening were exclusionary for participation in the study.

To screen for severe PTSD symptoms, the PCL-M [38] was used. Potential participants completed this questionnaire via the Qualtrics Research Suite Online Application as part of the online prescreening procedures. The PCL-M corresponds to criteria according to the fourth edition of the Diagnostic and Statistical Manual of Mental Disorders (DSM-IV [41]) and consists of 17 questions referring to military experiences and symptoms of stress and trauma (e.g., repeated, disturbing memories, thoughts, or images of a stressful military experience) answered on a rating scale of 1 (not at all) to 5 (extremely). A score greater than or equal to 50 indicates a probable diagnosis of PTSD according to DSM-IV criteria [42].

If potential participants scored 50 or greater on the PCL-M during online prescreening, the past-month version of the CAPS-5 [39] was performed at the in-person screening visit by a member of the UAB Office of Psychiatric Clinical Research. The 30-item structured interview evaluates PTSD diagnostic status and symptom severity by assessing onset and duration of symptoms, levels of distress, changes in social and occupational functioning, response validity, and symptoms of the dissociative subtype of PTSD. Each CAPS-5 item is rated on frequency and intensity, which is then combined into a single severity score for that symptom. A severity rating of 2 (moderate/threshold) indicates a symptom that meets diagnostic threshold for current PTSD [43]. If a potential participant met criteria for current PTSD, they were excluded from the study.

### 2.5. Main Outcome Measures

The Qualtrics Research Suite Offline Application (Qualtrics, Provo, UT, USA) was used for daily symptom reporting. As part of the daily report, participants scored symptoms on a 0–100 digital visual analog scale (VAS). GWI symptom severity, the primary outcome variable of interest, was assessed by asking, “Overall, how severe have your symptoms been today?” anchored on the left by, “No symptoms at all,” and on the right by, “Severe symptoms.” The single-item, overall GWI severity measure was chosen as the primary outcome, because of the multisymptom and idiosyncratic nature of the condition’s manifestation in affected individuals. Veterans living with GWI may primarily experience pain, fatigue, gastrointestinal distress, or any of several other common symptoms. The overall severity measure was determined to be a way to provide the most universal assessment of GWI symptom severity across participants with varying symptom presentations. Participants were instructed to use the GWI symptom severity measure to include whichever specific symptoms they attributed to GWI.

### 2.6. Secondary Outcome Measures

Several specific symptoms were assessed with similar one-item measures. These symptoms included pain, fatigue, cognitive dysfunction, depressed mood, dermatologic complaints, respiratory problems, and gastrointestinal distress. Of these, two (pain and fatigue) were endorsed by a sufficient number of participants to allow for statistical analysis. These two symptoms were measured with two separate 0–100 VAS items. Pain was assessed by asking, “Overall, how severe is your pain?” anchored on the left by, “No pain at all,” and on the right by, “Severe pain.” Fatigue was assessed by asking, “How fatigued have you felt today?” anchored on the left by, “Not fatigued at all,” and on the right by, “Severely fatigued.”

### 2.7. Statistical Analyses

Analyses were conducted using SPSS Statistics for Windows, version 24 (IBM Corp., Armonk, NY, USA). Effects of botanicals on symptom severity were tested using separate two-level linear mixed models (LMM). LMMs are designed for nested data structures and allow for the dependence between observations typically seen with repeatedly measured variables. In this study, longitudinal outcome assessments are nested within individuals and conditions, violating the assumption of independence between observations. For each botanical, three models were built with condition (baseline, placebo, lower-dose, and higher-dose) entered as a fixed factor and symptom severity (GWI, pain, or fatigue) as the dependent variable, resulting in a total of nine models. Subject ID was used as the subject identifier, and the day in the study was the repeated measures index. A compound symmetry repeated measures covariance structure was selected, as it improved the Bayesian information criterion (BIC) and Akaike information criterion (AIC) for model performance beyond the AR (1) autoregressive covariance structure. For main analyses, daily GWI symptom severity was entered as the dependent variable. For all analyses, the outcome variable was derived by taking a mean of the last 14 days of daily symptom severity reports for each participant during each condition (placebo, lower dose, higher dose). This was consistent with the registered analysis plan, in order to allow the botanicals time to exert clinical effects. A restricted maximum likelihood (REML) estimation approach was used. Post hoc contrasts were carried out with the least-squares differences method. Significance for all tests was set at *p* < 0.05.

Sample size was determined with a-priori power analyses conducted in G*Power 3 [44]. The botanical trials were powered to detect a medium effect (Cohen’s d = 0.5) with 0.99 power at a *p* = 0.05 level of significance. Ten individuals were needed to reach 0.99 power, due to the large number of repeated outcome assessments (56 per participant, repeated measures correlation of 0.5). The trials were not powered to detect small (Cohen’s d = 0.25) effects, with only 0.42 predicted power. Small effects, however, were not expected to have important clinical impacts on GWI.

## 3. Results

### 3.1. Participants

**Figure 2:**
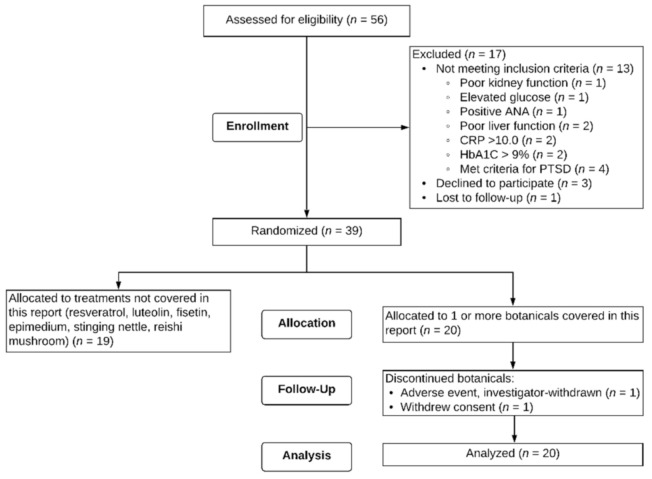
CONSORT Flow diagram. Twenty individuals were randomized to at least one of the treatments covered in this report.

Fifty-six male Veterans signed consent forms to participate in the study. Out of the 56 potential participants, one was lost to follow-up after the screening visit, 13 were excluded based on screening criteria, and three self-withdrew prior to starting capsules. Thirty-nine participants were eligible for the study and randomized to receive botanicals. Twenty individuals aged 47 to 65 (M = 51.4, SD = 4.62) received at least one of the three botanicals described in this paper. Figure 2 depicts a flow chart of study recruitment, enrollment, and attrition. Of these 20 individuals, 12 were assigned to only one of the three botanicals in this report, six were assigned to two of the three, and two were assigned to all three botanicals. Regarding order of botanical administration, for curcumin, participants were randomized to receive it 1st (*n* = 2), 2nd (*n* = 4), 3rd (*n* = 1), and 5th (*n* = 3). For boswellia, participants were randomized to receive it 1st (*n* = 5), 2nd (*n* = 1), 3rd (*n* = 2), 5th (*n* = 1), and 6th (*n* = 1). For maritime pine, participants were randomized to receive it 1st (*n* = 1), 2nd (*n* = 5), 3rd (*n* = 2), and 4th (*n* = 2). Seventeen (85%) of the participants identified as non-Hispanic White, two (10%) as non-Hispanic Black, and one (5%) as Hispanic. All 20 participants participated for a minimum of the baseline period (30+/−3 days) and one full round of capsules for a given botanical (placebo, lower-dose, higher-dose). Two participants did not complete their allocated botanical assignments. One participant was investigator-withdrawn because of elevated liver enzymes (ALT 123.0 Units/L, AST 49.0 Units/L) after 28 days of maritime pine. The participant was subsequently physician-diagnosed with a liver condition. This condition was not attributed to the study botanical, but the botanical was stopped in order to reduce the metabolic load on the liver. Another participant withdrew partway through the study due to an unexpected family emergency.

### 3.2. Curcumin

**Figure 3:**
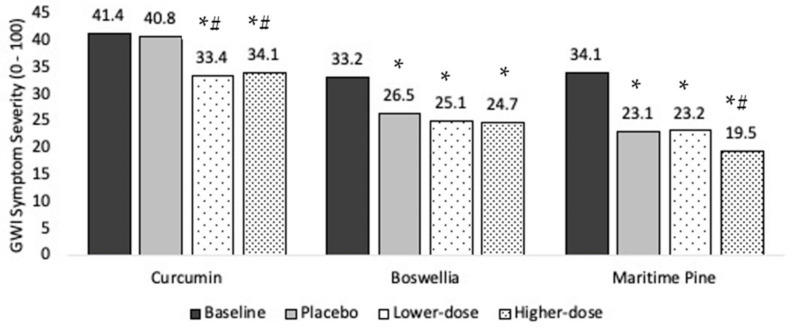
Main treatment effects of curcumin, boswellia, and maritime pine on Gulf War Illness (GWI) symptom severity. Average symptom levels (0–100) are presented for the baseline, placebo, lower-dose, and higher-dose conditions. * significantly lower than baseline (*p* < 0.05). *# significantly lower than baseline and placebo (*p* < 0.05).

Ten individuals completed the protocol. Baseline symptom severity was 41.4 (SD = 17.5). Symptom severity was 40.8 during placebo, 33.4 in lower-dose curcumin, and 34.1 in higher-dose curcumin, representing a 1.5%, 19.4%, and 17.6% drop in severity, respectively (Figure 3)

The LMM showed a significant main effect for condition (F (3, 558) = 12.0, *p* < 0.0001), indicating that participant symptom severity differed as a function of condition (i.e., baseline, placebo, lower-dose, higher-dose). Parameter estimates for each condition in the curcumin model were baseline M = 40.7 (SE = 5.1, df = 9.3, 95% CI (29.3, 52.1)), placebo M = 38.5 (SE = 5.1, df = 9.5, 95% CI (27.1, 50.0)), lower-dose curcumin M = 33.1 (SE = 5.1, df = 9.5, 95% CI (21.6, 44.5)), and higher-dose curcumin M = 34.7 (SE = 5.1, df = 9.5, 95% CI (23.2, 46.1)). Post hoc contrasts revealed that placebo was not significantly different from baseline (*p* = 0.797), but both lower-dose and higher-dose curcumin showed a significant reduction in symptom severity from baseline (*ps* < 0.0001). Both lower-dose and higher-dose curcumin also reduced overall GWI symptom severity over placebo (*p* < 0.0001 and *p* = 0.0003, respectively). Higher-dose curcumin did not significantly reduce symptom severity over lower-dose curcumin (*p* = 0.686).

### 3.3. Boswellia

Ten individuals completed the protocol. Baseline overall symptom severity was 33.2 (SD = 19.5). Symptom severity was 26.5 during placebo, 25.1 in lower-dose boswellia, and 24.7 in higher-dose boswellia, representing a 20.1%, 24.4%, and 25.7% drop in severity, respectively (Figure 3).

The LMM showed a significant main effect for condition (F (3, 589) = 37.2, *p* < 0.0001). Parameter estimates for each condition in the boswellia model were baseline M = 32.0 (SE = 4.1, df = 9.3, 95% CI (22.7, 41.3)), placebo M = 20.2 (SE = 4.2, df = 9.8, 95% CI (10.9, 29.6)), lower-dose boswellia M = 19.7 (SE = 4.2, df = 9.8, 95% CI (10.4, 29.1)), and higher-dose boswellia M = 20.3 (SE = 4.2, df = 9.8, 95% CI (11.0, 29.7)). Post hoc contrasts showed that all conditions reduced reported GWI symptom severity from baseline (placebo: *p* < 0.0001; lower-dose boswellia: *p* < 0.0001; higher-dose boswellia: *p* < 0.0001). However, neither lower-dose nor higher-dose boswellia reduced overall GWI symptom severity over placebo (lower dose: *p* = 0.726; higher dose: *p* = 0.869).

### 3.4. Maritime Pine

Ten individuals completed the protocol. Baseline overall symptom severity was 34.1 (SD = 20.1). Symptom severity was 23.1 during placebo, 23.2 in lower-dose maritime pine, and 19.5 in higher-dose maritime pine, representing a 32.3%, 32.0%, and 42.8% drop in severity, respectively (Figure 3).

The LMM showed a significant main effect for condition (F (3, 635) = 71.5, *p* < 0.0001). Parameter estimates for each condition in the maritime pine model were baseline M = 34.5 (SE = 4.7, df = 10.2, 95% CI (24.2, 44.9)), placebo M = 24.9 (SE = 4.7, df = 10.6, 95% CI (14.5, 35.4)), lower-dose maritime pine M = 21.6 (SE = 4.7, df = 10.6, 95% CI (11.1, 32.0)), and higher-dose maritime pine M = 19.9 (SE = 4.7, df = 10.8, 95% CI (9.4, 30.4)). All conditions reduced GWI symptom severity from baseline (all *ps* < 0.0001). Lower-dose maritime pine was not significantly different from placebo (*p* = 0.954). Higher-dose maritime pine was significantly different from both placebo (*p* = 0.006) and lower-dose maritime pine (*p* = 0.005).

### 3.5. Secondary Outcomes

Effects of the three botanicals on pain and fatigue severity are presented in Table 1. Lower-dose curcumin was significantly better than placebo at reducing fatigue. Boswellia was not better than placebo at reducing pain or fatigue. Lower-dose maritime pine was better than placebo at reducing pain, and higher-dose maritime pine was better than placebo at reducing both pain and fatigue.

### 3.6. Adverse Events

Of the 20 total participants, 10 participants reported adverse events (AEs) that could potentially be attributed to the study botanicals. All reported AEs were mild to moderate in severity. The most commonly reported side effects were worsening fatigue, diarrhea, worsening gastrointestinal complaints, and migraine headaches. Two participants reported worsening fatigue across all conditions throughout their participation. Adverse events for botanicals included in this report are summarized in Table 2.

## 4. Discussion

In this clinical trial, we investigated the effects of curcumin, boswellia, and maritime pine on symptoms of GWI. We found significant reduction in GWI symptom severity for both lower- and higher-dose curcumin, as well as for higher-dose maritime pine. There were no significant reductions in GWI symptom severity seen for boswellia. Each of the botanical compounds was found to be well-tolerated at the tested dosages, and no clinically relevant adverse events attributed to the botanicals were observed.

This unique, multibotanical trial approach was employed in order to rapidly screen several potential treatments for GWI. This approach allowed us to conduct nine small clinical trials in a three-year period, with the goal of prioritizing potential treatments for future efficacy studies. As these are trials with small sample sizes, the results are preliminary, and no treatment recommendations can be made based on the results.

Curcumin has been proposed as a treatment for GWI with some animal research supporting its use [16]. We are not aware of any clinical trials testing the effectiveness of curcumin in individuals with GWI or potentially related conditions such as fibromyalgia or myalgic encephalomyelitis/chronic fatigue syndrome (ME/CFS). In this study, we found that GWI symptoms were significantly lower in both lower- and higher-dosage curcumin than in placebo. However, the participants in the curcumin condition showed a smaller placebo response than is typical for clinical trials, even compared with other compounds in the current study. There were no anomalies in the dataset, and it is unknown why the placebo response was small; though, it may be related to the experience of trialing multiple therapeutic agents in a short period of time.

We are not aware of any published clinical trials examining boswellia for efficacy in GWI or other chronic, multisymptom illnesses, such as fibromyalgia or ME/CFS. The agent has been proposed as a treatment for neuroinflammatory disorders, given its ameliorating effects on endotoxin-induced inflammatory responses [45]. We found no evidence that the treatment, given daily at 400 mg or 800 mg, is helpful for GWI.

Likewise, we have not seen clinical trials for maritime pine in GWI, or related conditions such as fibromyalgia or ME/CFS. While there is limited evidence of a central anti-inflammatory and neuroprotective role from animal studies [31], human studies are lacking. We found that the higher dosage of maritime pine extract (400 mg per day) was associated with a significant drop of GWI symptoms over placebo.

Curcumin, boswellia, and maritime pine were previously identified as having among the greatest treatment effect on osteoarthritis pain across 20 supplements reviewed [46]. While all three botanicals have been observed to possess anti-inflammatory properties, their active agents are different [47], and it was not expected they would all impact GWI similarly. Curcumin’s association with decreased fatigue and maritime pine’s association with decreased pain and fatigue may further suggest anti-inflammatory effects of these agents. It is also possible that the botanicals included in this report act via other mechanisms, including neurogenesis (curcumin and boswellia [16,48]) or antioxidative actions (curcumin, boswellia, and maritime pine [48,49,50]). However, to more definitively explore mechanisms of clinical effects, blood-based markers of inflammation should be examined.

### Limitations

A concern with all botanical trials is the purity of the compound(s) used. Most regulation of complementary and alternative treatments is optional, and it can be difficult to verify the quality of the product. In many cases, there may be dozens of sourcing options available, and research (on boswellia, for example) has found great variability in the purity of available products [51]. Differences in source, dosage, purity, ratio of components, additive ingredients, and other factors may modulate the effectiveness of a compound purchased by an individual seeking symptom relief. Another issue common to all trials is that beneficial effects may be closely tied to dosage, and most trials can only test one dosage. Because of the lack of evidence indicating which dosages of these botanical compounds might be effective to provide symptom relief, we tested two different dosages for each compound, following best practices for naturopathic medicine. Still, it is unknown whether lower or higher dosages than those tested in this preliminary trial would have been more effective at reducing GWI symptoms, or if dosage needs to be tailored to each individual based patient characteristics such as BMI. Further, in this study, the higher dose is conflated with treatment duration, as all participants had already been on one month of a lower dose therapy prior to initiating the higher dose of the same agent.

While the multiple botanical trial method employed here has advantages of economy, there are also problems associated with this condensed study format. In general, dedicating more resources to keeping a small number of participants enrolled for an extended period of time means fewer resources are devoted to the overall sample size. With small sample sizes comes the risk that the sample is not representative of the large population, and the findings may not hold when tested in another group. Generalizability of the study findings is especially limited in terms of sex, as only men were recruited. While women made up only 7% of military personnel in the 1991 Gulf War, and though it is possible they were more likely to develop GWI, a substantially greater number of men currently are living with GWI [52].

The lengthy study participation also required us to administer each botanical for only a short period of time (30 +/− 3 days at each dosage), in order to keep initial overall participation under a year. It is possible that one month is insufficient to observe the full potential effect of the compounds studied here. While we included a placebo period between botanicals to minimize interactions between test conditions, the study design still carried a risk of carryover effects, as only two weeks after concluding a higher-dose botanical, symptom reports were taken for the placebo condition of the next botanical. An additional potential shortcoming of this study design is the possibility of order effects. The small sample size and large number of possible orders prevented us from directly testing order effects. We attempted to mitigate possible order effects through the pseudo-random assignment of botanical conditions. Furthermore, across botanical conditions, placebo severity scores were highly consistent (Cronbach’s alpha = 0.895), suggesting little effect of order on symptom reporting. Despite those attempts, it is still possible that some botanicals affected responses to other, later botanical assignments. It is also possible that the study design allows for behavioral changes in symptom reporting over time. For example, expectancy of beneficial effects may change depending on whether a previous botanical within the study was perceived to be effective or not.

It is necessary to acknowledge that individuals in the curcumin group reported greater baseline symptom severity and greater placebo symptom severity than individuals taking other botanicals. Additionally, symptom severity for participants while on curcumin, though significantly lower than during baseline or placebo, was higher than symptom severity for participants on other botanicals. Given the small sample sizes, noticeable differences between treatment groups are expected. The majority of curcumin participants (6/10) did not receive boswellia or maritime pine. However, it is unknown whether the differences in the curcumin group may have resulted in differences in response to treatment.

Because of the small sample size, it was not possible to conduct any type of statistical analysis on the reported adverse events. No side effects were endorsed by more than one participant for any given study condition. The placebo period had as many endorsed side effects (9) as lower-dose (9) and higher-dose (10) medications. While there is no indication that these botanicals have expected side effects at these dosages and in this population, considerably larger sample sizes would be needed to make reliable conclusions about adverse effects. Furthermore, our analyses did not implement a *p*-value correction for multiple comparisons (e.g., Bonferroni), as it was not part of our registered analysis plan. As a result, the chance of false positive findings is increased; however, a correction for the three main tests would result in a *p*-value threshold of 0.017, which does not change the results of the tests for main effects in our models.

An important final caveat is that this study did not make use of a validated GWI outcome measure. The single-item was designed as a low-burden method of assessing GWI severity daily and a way of accommodating the idiosyncratic nature of symptom presentation. It is possible that our outcome lacked sensitivity or specificity in tracking GWI severity. With recent efforts to develop common GWI research tools, future projects should use validated outcomes when available.

## 5. Conclusions

Because Veterans living with GWI represent a fixed and aging cohort, focus must be placed on quickly identifying effective and easily accessible treatments for these individuals. While a specific and targeted pharmaceutical treatment for GWI would be preferred, it becomes less likely over time that pharmaceutical companies will put forward an FDA-approved treatment in time to improve the quality of life of Veterans living with GWI. Therefore, priority should be placed on screening currently available compounds (pharmaceutical, botanical, and other) to determine if they could be used to improve quality of life for these Veterans immediately. The novel treatment screening process described in this paper is one such tool for quickly and economically identifying potential treatments deserving further study. To our knowledge, these are the first tests of these three botanical agents for GWI in human participants. Using this approach, we have concluded that curcumin and maritime pine may be promising candidates for further study for the treatment of GWI.

## Figures and Tables

**Table 1:** Means (and standard deviations) and linear mixed models (LMM) results for the secondary outcomes of self-reported pain and fatigue. Results are presented separately for curcumin, boswellia, and maritime pine.

	Baseline	Placebo	Lower Dose	Higher Dose	LMM
**Curcumin**					
Pain	36.4 (23.3)	30.2 (19.2)	29.2 (17.8) *	33.0 (21.1)	F (3, 556) = 7.2, *p* = 0.0001
Fatigue	36.2 (22.2)	36.0 (22.3)	30.9 (19.9) *#	34.0 (20.2) *	F (3, 556) = 5.5, *p* = 0.001
**Boswellia**					
Pain	33.1 (20.3)	20.4 (14.1) *	18.6 (10.3) *	18.9 (12.6) *	F (3, 589) = 54.8, *p* < 0.0001
Fatigue	37.2 (22.9)	29.1 (22.5) *	30.3 (21.6) *	32.0 (21.5)	F (3, 589) = 6.2, *p* < 0.0001
**Maritime Pine**					
Pain	34.8 (20.4)	22.3 (17.0) *	19.6 (15.1) *#	19.9 (14.2) *#	F (3, 635) = 72.5, *p* < 0.0001
Fatigue	46.9 (21.4)	42.2 (24.5)	41.2 (21.5) *	38.8 (22.1) *#	F (3, 635) = 13.6, *p* < 0.0001

* = significantly lower than baseline, *# = significantly lower than baseline and placebo.

**Table 2:** Incidence of self-reported adverse events.

	Boswellia			Curcumin			Maritime Pine		
Adverse Event	P	LD	HD	P	LD	HD	P	LD	HD
Worsening fatigue	1	1	1	1	1	1	1	-	-
Diarrhea	-	-	1	1	1	-	-	1	-
Migraine	-	-	-	1	1	1	-	-	-
Worsening gas	1	1	-	-	-	-	-	-	-
Abdominal cramping	-	-	-	1	1	-	-	-	-
Flushing of the face	-	-	-	1	1	-	-	-	-
Dizziness	1	-	-	-	-	-	-	-	-
Increased anxiety	-	-	1	-	-	-	-	-	-
Worsening gas pain	-	-	-	-	-	1	-	-	-
Nausea	-	-	-	-	-	1	-	-	-
Itching	-	-	-	-	-	1	-	-	-
Severe headache	-	-	-	-	-	1	-	-	-
Worsening GERD	-	-	-	-	-	1	-	-	-
Neck stiffness	-	-	-	-	-	-	-	1	-

P = placebo, LD = lower dose, HD = higher dose, GERD = gastroesophageal reflux disease.

## Data Availability

The datasets used and/or analyzed during the current study are available from the corresponding author upon reasonable request and the approval of the data owner.
